# Short-chain L-3-hydroxyacyl-CoA dehydrogenase: A novel vital oncogene or tumor suppressor gene in cancers

**DOI:** 10.3389/fphar.2022.1019312

**Published:** 2022-10-14

**Authors:** He Fang, Hanyang Li, Hang Zhang, Shu Wang, Shuang Xu, Li Chang, Yongsheng Yang, Ranji Cui

**Affiliations:** ^1^ Department of Hepatobiliary and Pancreatic Surgery, The Second Hospital of Jilin University, Changchun, China; ^2^ Department of Thyroid Surgery, The Second Hospital of Jilin University, Changchun, China; ^3^ Department of Radiotherapy, The Second Hospital of Jilin University, Changchun, China; ^4^ Department of Anesthesiology, The Second Hospital of Jilin University, Changchun, China; ^5^ Department of Pathology, The Second Hospital of Jilin University, Changchun, China; ^6^ Jilin Provincial Key Laboratory on Molecular and Chemical Genetic, The Second Hospital of Jilin University, Changchun, China

**Keywords:** HADH, oncogene, tumor suppressor gene, differential expression, tumor microenvironment, tumor-infiltrating immune cells

## Abstract

The reprogramming of cellular metabolism is frequently linked to tumorigenesis. Glucose, fatty acids, and amino acids are the specific substrates involved in how an organism maintains metabolic equilibrium. The *HADH* gene codes for the short-chain L-3-hydroxyacyl-CoA dehydrogenase (HADH), a crucial enzyme in fatty acid oxidation that catalyzes the third phase of fatty acid oxidation in mitochondria. Increasing data suggest that HADH is differentially expressed in various types of malignancies and is linked to cancer development and progression. The significance of HADH expression in tumors and its potential mechanisms of action in the onset and progression of certain cancers are summarized in this article. The possible roles of HADH as a target and/or biomarker for the detection and treatment of various malignancies is also described here.

## 1 Introduction

Cancer is a major public health problem worldwide and has been identified as the biggest obstacle to improving life expectancy in the 21st century, making it a continuous focus of scientific attention (Fitzgerald et al., 2021). Cancer remains one of the leading causes of death (Galván Morales et al., 2020), and by 2018, approximately more than 11 million people have been diagnosed with cancer (Wang et al., 2018). The American Cancer Society expects that number to increase further, with an estimated 1,918,030 new cases and 609,360 cancer-related deaths occurring in the United States in 2022 (Siegel et al., 2022). More than 4 million new cancer patients and more than 2 million cancer-related mortalities are reported every year in China. Although cancer is treated in a variety of ways, such as surgery, chemotherapy, radiation therapy, and targeted therapy, 3- and 5-year cancer-specific survival rates remain poor (Kim and Kim, 2015; Vijayvergia et al., 2015; Koinis et al., 2016; Miller et al., 2016; Nakashima, 2018; Zhang and Zhang, 2018; Wen et al., 2019). Despite cancer-related deaths having declined overall (Henley et al., 2020), it is important to note that this decrease is mainly a result of early detection and prevention rather than better treatments (Etzioni et al., 2003; Chabner et al., 2005; Huff et al., 2006; Buskwofie et al., 2020). The vast majority of cancers are asymptomatic in their early stages of development (Smith et al., 2007; Zheng et al., 2020). Therefore, it is very important to explore the mechanisms of tumorigenesis and development, search for new diagnostic and prognostic markers, and develop effective and novel therapeutic methods. Further advancements will have a major impact on improving cancer patient survival rates.

Numerous studies have strongly demonstrated that specific genes, such as oncogenes and tumor suppressor genes, are risk factors for certain malignancies (Khan et al., 2018; Elek et al., 2020; Shareefi et al., 2020; Vysotskaia et al., 2020; Xiong et al., 2020). Oncogenes can promote tumor growth when they are activated, whereas tumor suppressor genes hinder tumor growth and development. Oncogene-directed metabolic reprogramming (Ward and Thompson, 2012), which appears to be a common hallmark of highly malignant tumors (Hanahan and Weinberg, 2011) regardless of their carcinogenic origin (Bustamante et al., 1981), is the most prevalent cause of metabolic alterations.

The tumor microenvironment (TME) is involved with carcinogenesis in a complex manner and can influence cancer incidence and progression (Arneth, 2019). The non-malignant cells in the TME frequently play a key role in all phases of tumorigenesis by stimulating and promoting uncontrolled cell proliferation (Balkwill et al., 2012; Hanahan and Coussens, 2012). The extracellular matrix (ECM), blood vessels, fibroblasts, lymphocytes, signaling chemicals and bone marrow-derived inflammatory cells make up the TME (Spill et al., 2016; Del Prete et al., 2017). Immune cells, such as lymphocytes, macrophages, and granulocytes, are a very significant component and can impact the formation, growth, and development of tumor cells in patients with various forms of cancer (Mantovani et al., 2008; Grivennikov et al., 2010; Hanahan and Weinberg, 2011; Lebleu, 2015; Spill et al., 2016; Del Prete et al., 2017). The TME homeostasis is the result of numerous complex interactions, many of which involve cell metabolism (Nieman et al., 2011; Colegio et al., 2014; Chang et al., 2015; Sousa et al., 2016; Chen P. et al., 2017; Angelin et al., 2017; Buck et al., 2017; Bantug et al., 2018; Zhang et al., 2018; Vitale et al., 2019a; Vitale et al., 2019b). Under normal circumstances, an organism’s metabolism is in equilibrium. The reprogramming of cellular metabolism is a hallmark of tumorigenesis (Hanahan and Weinberg, 2011; Wettersten et al., 2017) and aids in the conversion of large amounts of nutrients into cellular building blocks such as nucleotides, amino acids, and lipids (Ren et al., 2020), resulting in an excess of the antioxidant glutathione to produce new cells (Gao et al., 2009; Hirschhaeuser et al., 2011). Glucose, fatty acids, and amino acids are the substrates that keep metabolic homeostasis in check (Heslegrave and Hussain, 2013). Fatty acid metabolism is often altered in cancer cells to sustain cell proliferation, meet energy needs, and produce metabolites for anabolic activities (Currie et al., 2013; Sanchez and Simon, 2018). Several reports have shown that the enzymes involved in fatty acid β-oxidation are reduced in individuals with malignancies (Tanaka et al., 2013; Enjoji et al., 2016). β-oxidation in mitochondria breaks down fatty acids (Bartlett and Eaton, 2004), and this is a crucial metabolic process for energy balance in organs such the liver, heart, and skeletal muscle (Heslegrave and Hussain, 2013). Enoyl-CoA hydratase, acyl-CoA dehydrogenase, ketoacyl-CoA thiolase, and hydroxyacyl-CoA dehydrogenase are four main enzymes involved in the breakdown of fatty acids (Shen et al., 2017). The role of fatty acids in the breakdown of cancer cells, however, is still debated. Additional research is required to further understand how fatty acid metabolism reprogramming influences the formation and development of cancers.

The human HADH gene, which has 10 exons and is expressed in most tissues, is found on chromosome 4q25 (Heslegrave and Hussain, 2013). The gene encodes the intramitochondrial homodimer enzyme short-chain-L-3-hydroxyacyl-CoA dehydrogenase (HADH), which is a key enzyme in the third step of fatty acid β-oxidation (Vredendaal et al., 1996; Eaton et al., 2000; Yang et al., 2005; Kapoor et al., 2010; Schulz et al., 2011; Popa et al., 2012; Arya et al., 2014; Jiang et al., 2021). During extended fasting, HADH transforms short- and medium-chain fatty acids into ketones to fuel the liver, heart, muscles, and pancreas (Shen et al., 2017), with enzyme activity being highest in the pancreas and especially in the islets of Langerhans (Agren et al., 1977). Several investigations have shown that HADH plays an important role in controlling insulin secretion from the β-cell (Hardy et al., 2007; Martens et al., 2007; Filling et al., 2008; Li et al., 2010; Heslegrave et al., 2012) and that inhibiting its activity results in a considerable increase in insulin secretion (Kapoor et al., 2009; Heslegrave et al., 2012; Heslegrave and Hussain, 2013).

**FIGURE 1:**
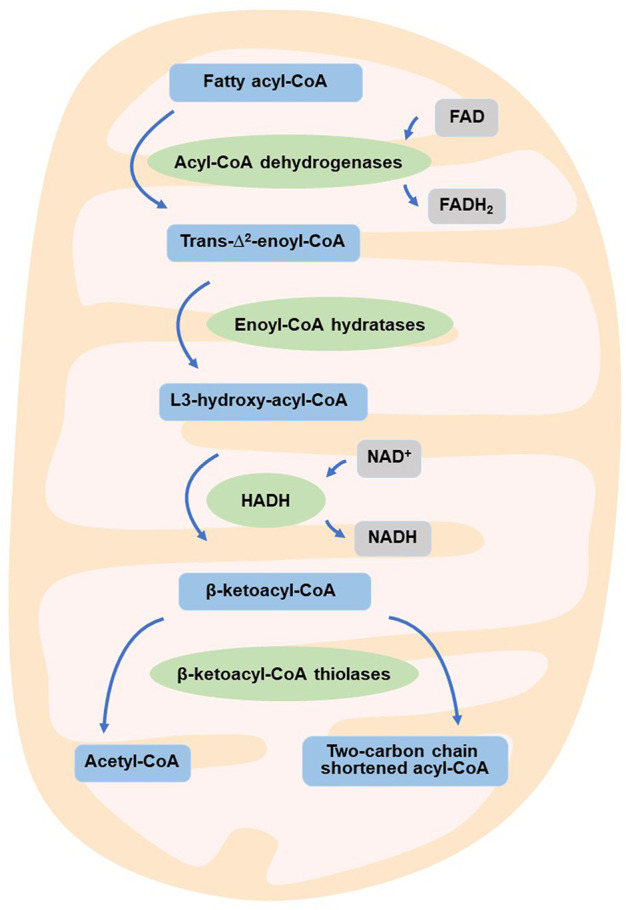
HADH is involved in fatty acid β-oxidation.

**FIGURE 2:**
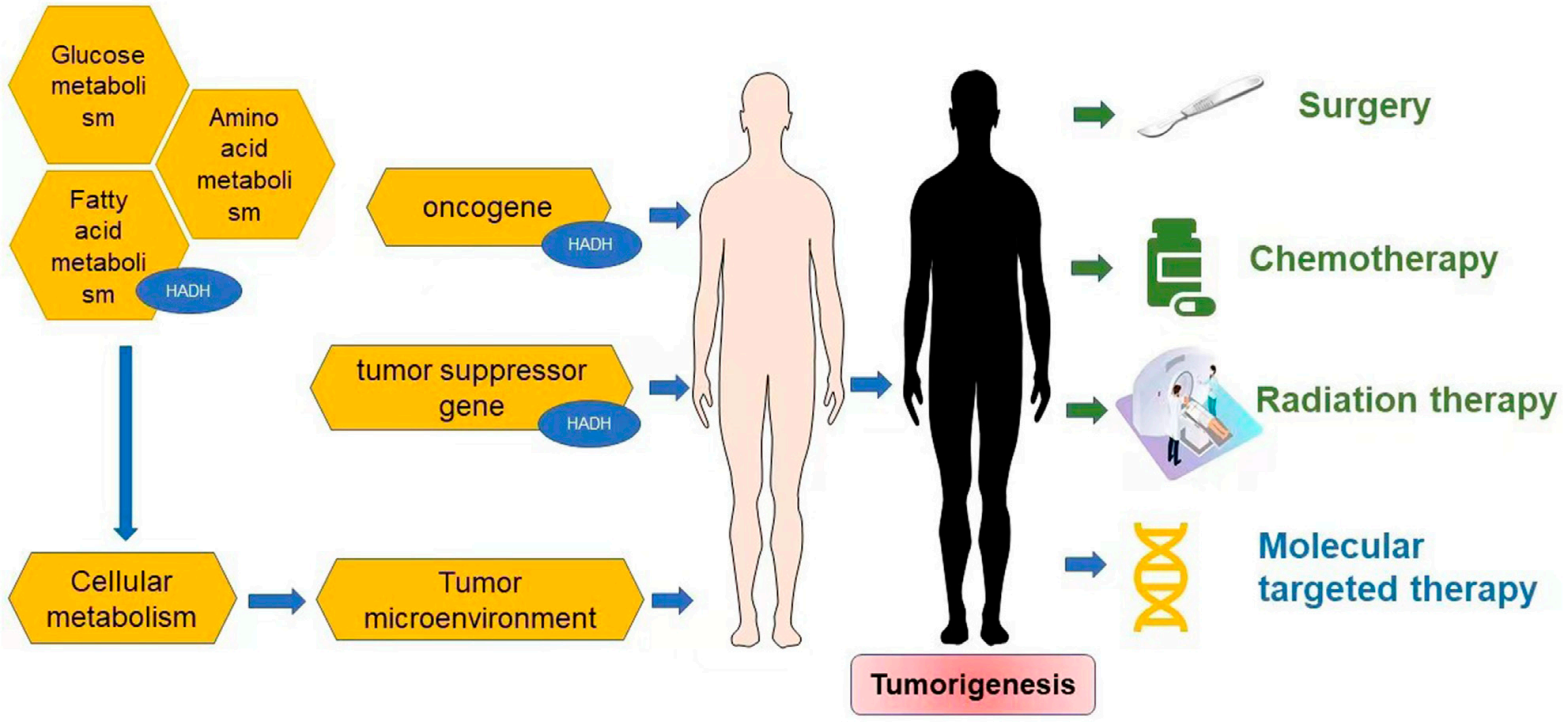
Cancer progression and therapy.

Reprogramming of energy metabolism is a well-known feature of malignancies (Hanahan and Weinberg, 2011; Wettersten et al., 2017), and fatty acid metabolism is also thought to be a crucial contributor to cancer cell proliferation (Currie et al., 2013). HADH is a crucial enzyme in the oxidation of fatty acids (Flanagan et al., 2013; Babiker et al., 2015; Çamtosun et al., 2015; Satapathy et al., 2016; Boerrigter-Eenling et al., 2017). Dehydrogenation, hydration, dehydrogenation again, and thiolytic cleavage are the four enzymatic processes that make up fatty acid oxidation (FAO) (Wanders et al., 1999). HADH is a component of the enzymatic reaction mentioned above (Houten and Wanders, 2010). In the mitochondrial matrix, HADH catalyzes the penultimate process in the β-oxidation of fatty acids (Vredendaal et al., 1998), dehydroxylating medium- and short-chain NAD^+^-dependent L3-hydroxy-acyl-CoA to produce β-ketoacyl-CoA and NADH, respectively (Figure 1) (Houten et al., 2016). The expression levels of HADH are also higher than those of other fatty acid β oxidases, such as acyl-CoA dehydrogenase and acetyl-CoA acyltransferase 2. Additionally, HADH enzymatic activity is the most effective for metabolizing medium-chain length fatty acids (Pepin et al., 2010). Reduced HADH expression can impede β-oxidation and stimulate fatty acid buildup, which leads to fatty acid metabolism reprogramming and promotes tumor development (Wettersten et al., 2017). Growing evidence has recently shown its importance in the occurrence and progression of several malignancies (Shen et al., 2017; Wilkins et al., 2017; Nwosu et al., 2018; Voloshanenko et al., 2018; Ren et al., 2020; Jiang et al., 2021; Sun et al., 2022). HADH has been identified as a possible target for the diagnosis and therapeutic treatment of many malignancies because of this apparent influence on carcinogenesis (Figure 2). The specific functions and molecular details of HADH in the incidence and progression of various cancers are summarized in this article, with an emphasis on gastric cancer, kidney renal clear cell carcinoma, liver cancer, colon cancer, and acute myeloid leukemia.

## 2 Short-chain L-3-hydroxyacyl-CoA dehydrogenase in cancers

HADH expression is upregulated or downregulated in different types of cancers, including gastric cancer, kidney renal clear cell carcinoma, liver cancer, colon cancer, and acute myeloid leukemia. The relevant clinicopathological features and molecular mechanisms of HADH in these cancers are summarized in Table 1 and detailed in the rest of this section.

**TABLE 1:** Functional characteristics and clinical features of HADH in human cancers.

Cancer types	Expression	Role	Functional role	Related genes	Clinical features	References
Gastric cancer	Downregulated	Tumor suppressor gene	Inhibits proliferation, migration, and invasion	PTEN, AKT	Early clinical stage, long OS and DFS, and good clinical prognosis	(Shen et al., 2017; Du et al., 2021)
Kidney renal clear cell carcinoma	Downregulated	Tumor suppressor gene	No studies have been reported	genes in EMT, TNF- α, IL-6-JAK-STAT3, and interferon-γ signaling pathway	Early clinical stage, low histologic grade, long OS and DFS, low distant metastasis, good prognosis, and immune infiltration	(Zhang et al., 2019; Jiang et al., 2021; Sun et al., 2022)
Liver cancer	Downregulated	Tumor suppressor gene	No studies have been reported	No studies have been reported	No studies have been reported	Nwosu et al. (2018)
Colon cancer	Upregulated	Oncogene	Promotes proliferation	Wnt5a/b, Evi/Wls, RoR2, Dvl2, ATF2 and ATF4	Poor clinical outcome	(Voloshanenko et al., 2018; Ren et al., 2020)
Acute myeloid leukemia	Upregulated	Oncogene	No studies have been reported	GPX-7 and RPP40	Poor OS	Wei et al. (2020)

PTEN, phosphatase and tensin homolog; AKT, serine threonine kinase; EMT, epithelial-mesenchymal transition; TNF- α, tumor necrosis factor- α; IL-6, interleukin 6; JAK, janus kinase; STAT3, signal transducer and activator of transcription 3; ROR2, receptor tyrosine kinase like orphan receptor 2; Dvl2, disheveled segment polarity protein 2; ATF2, activating transcription factor 2; ATF4, activating transcription factor 4; GPX-7, glutathione peroxidase 7; Rpp40, ribonuclease *p* protein subunit p40; OS, overall survival; DFS, disease-free survival.

### 2.1 Gastric cancer

#### 2.1.1 Functional characteristics and clinical features of short-chain L-3-hydroxyacyl-CoA dehydrogenase in gastric cancer

HADH is expressed at lower levels in gastric cancer tissues compared with that in normal gastric tissues. Shen et al. (2017) showed that downregulation of HADH was significantly correlated with advanced clinical stage, low overall survival (OS), low disease-free survival (DFS), and poor clinical prognosis. Hence, HADH expression has been proposed as an independent prognostic factor that affects patient survival rates. Mechanistically, reduced HADH expression significantly promoted cell proliferation and increased migration and invasion of tumor cells *in vitro*; in contrast, overexpression of HADH inhibited the proliferation of gastric cancer cells (Shen et al., 2017).

In summary, HADH is a potential novel tumor suppressor gene in gastric cancer that can inhibit cell proliferation, migration, and invasion of gastric cancer cells. Furthermore, its expression levels are correlated with cancer progression and patient survival.

#### 2.1.2 Signaling pathways influenced by short-chain L-3-hydroxyacyl-CoA dehydrogenase in gastric cancer

**FIGURE 3:**
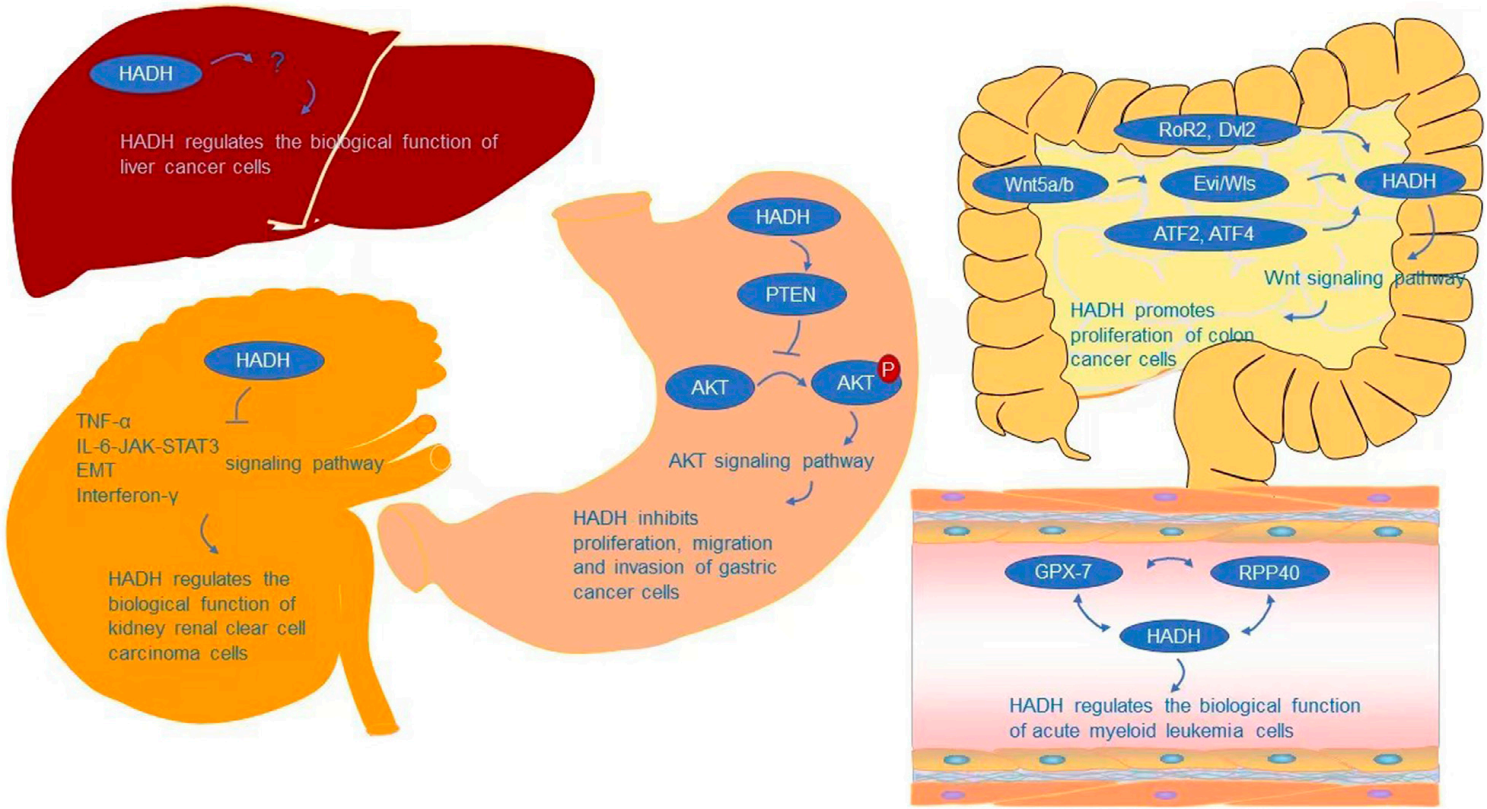
Underlying molecular mechanisms of HADH in cancers.

The AKT signaling pathway is required for HADH to regulate cell proliferation, migration, and invasion, according to Shen et al. (Shen et al., 2017). AKT signaling is a growth-regulating biological pathway that has been shown to improve tumor cell survival, proliferation, and motility in a variety of tumor types (Chan et al., 2014; Bao et al., 2021; Junaid et al., 2021; Tsai et al., 2021). AKT, also known as protein kinase B (PKB), is a critical node in many signaling pathways, as well as one of the most essential and flexible protein kinases in human physiology and illness (Meier and Hemmings, 1999; Manning and Cantley, 2007; Revathidevi and Munirajan, 2019). Many interesting advancements in the mechanism controlling AKT activity have been achieved since its identification as an oncogene homologue of murine leukemia virus AKT8 (Staal, 1987; Bellacosa et al., 1991) and protein kinase C (Jones et al., 1991). As a critical regulatory protein of cell growth, survival, proliferation, and metabolism (Kennedy et al., 1997; Sun et al., 2001; Vivanco and Sawyers, 2002; Bellacosa et al., 2005; Manning and Cantley, 2007; Broustas et al., 2012; Sanidas et al., 2014; Fan et al., 2018; Li et al., 2021), AKT crosses multiple signaling pathways (Gao and Pan, 2001; Ward et al., 2011; Yecies et al., 2011; Yin et al., 2017) and participates in a range of physiological activities. AKT is involved in the development of a variety of human malignancies (Manning and Cantley, 2007; Revathidevi and Munirajan, 2019). AKT gene mutations are uncommon, while AKT gene amplification and overexpression are widespread in malignancies such as gastric, colon, liver, thyroid, and ovarian tumors (Staal, 1987; Bellacosa et al., 1995; Cheng et al., 1996; Nakatani et al., 1999; Roy et al., 2002; Knobbe and Reifenberger, 2003; Xu et al., 2004; Altomare and Testa, 2005; Parsons et al., 2005; Carpten et al., 2007; Malanga et al., 2008; Mohamedali et al., 2008; Shoji et al., 2009; Zilberman et al., 2009; Askham et al., 2010; Mundi et al., 2016; Manning and Toker, 2017). Overexpression and activation of AKT have been linked to the initiation or progression of a number of human malignancies (Samuels et al., 2004; Cully et al., 2006; Cerami et al., 2012; Fruman and Rommel, 2014). AKT has a role in several physiological processes and, once activated, can affect the activity of several downstream proteins that control cell growth, survival, proliferation, and metabolism (Kennedy et al., 1997; Sun et al., 2001; Vivanco and Sawyers, 2002; Bellacosa et al., 2005; Hay, 2005; Manning and Cantley, 2007; Broustas et al., 2012; Sanidas et al., 2014; Liu H. W. et al., 2018; Fan et al., 2018). An abnormal loss or increase of AKT activation underpins the pathogenesis of a variety of complicated illnesses, including type 2 diabetes and cancers (Manning and Cantley, 2007). AKT also affects cell survival by phosphorylating and activating a number of oncoproteins implicated in cell cycle progression and carcinogenesis, including murine double minute (MDM2), S-phase kinase-associated protein 2 (Skp2), IKKα, and E3 ligase (Datta et al., 1999; Ozes et al., 1999; Mayo and Donner, 2001; Zhou et al., 2001; Chan et al., 2012; Chan et al., 2014). PTEN is a tumor suppressor that causes a significant reduction in cell proliferation by arresting the cell cycle in the G1 phase. PTEN and PHLPP2 are the most important negative regulators of AKT (Stambolic et al., 1998; Meng et al., 2007). Therefore, PTEN inactivation can potentially activate AKT by promoting AKT’s phosphorylation ability, leading to a cell survival advantage and uncontrolled cell proliferation (Downward, 2003; Gomes et al., 2014; Liu J. et al., 2018). Shen et al. (Shen et al., 2017) showed that downregulation of HADH could inhibit the expression of PTEN and promote the phosphorylation of AKT, further stimulating the proliferation, migration, and invasion of gastric cancer cells by activating the AKT pathway (Figure 3).

### 2.2 Kidney renal clear cell carcinoma

#### 2.2.1 Functional characteristics and clinical features of short-chain L-3-hydroxyacyl-CoA dehydrogenase in kidney renal clear cell carcinoma

Similar to gastric cancer tissues, HADH expression was markedly downregulated in kidney renal clear cell carcinoma tissues compared with that in adjacent non-cancerous tissues (Zhang et al., 2019; Jiang et al., 2021; Sun et al., 2022). Additionally, similarly to gastric cancer, HADH downregulation was significantly associated with poor OS, DFS, and poor prognosis in kidney renal clear cell carcinoma (Zhang et al., 2019; Jiang et al., 2021; Sun et al., 2022). It was positively correlated with the early clinical stage of disease and low histologic grade (Jiang et al., 2021; Sun et al., 2022). Moreover, HADH expression was also associated with tumor-infiltrating immune cells (TIICs) in kidney renal clear cell carcinoma (Jiang et al., 2021). Levels of M2 macrophages, naïve B cells, resting mast cells, and resting dendritic cells were positively correlated with HADH expression, while amounts of follicular helper T cells, plasma cells, regulatory T cells (Tregs), and neutrophils were negatively correlated with HADH expression (Jiang et al., 2021). These results suggest that HADH has an important role in the regulation of the immune microenvironment in kidney renal clear cell carcinoma.

In conclusion, HADH may be a novel tumor suppressor gene in kidney renal clear cell carcinoma, and its reduced expression is associated with immune cell infiltration and poor prognosis.

#### 2.2.2 Signaling pathways influenced by short-chain L-3-hydroxyacyl-CoA dehydrogenase in kidney renal clear cell carcinoma

Analyses of biological processes have indicated that HADH is associated with cell cycle arrest and negative regulation of the cell cycle (Zhang et al., 2019). Jiang et al. (2021) further analyzed this using GSEA, finding that the inflammatory response, TNF-α, IL-6-JAK-STAT3, epithelial-mesenchymal transition (EMT), and interferon-γ signaling pathways were activated in the HADH-low expression group, while fatty acid metabolism and protein secretion were inhibited (Figure 3). However, the specific molecular mechanism of how HADH can inhibit kidney renal clear cell carcinoma progression requires further study.

### 2.3 Liver cancer

Nwosu et al. (2018) found that HADH involved in fatty acid β-oxidation was expressed at lower levels in poorly differentiated hepatocellular carcinoma (HCC) cells compared with that in well differentiated HCC cells. However, whether the higher proliferation and migration rates of these poorly differentiated HCC cells are directly related to metabolic changes, including fatty acid β-oxidation, is still unknown. Further research is needed on the biological function of HADH and its signaling pathways in HCC (Figure 3).

### 2.4 Colon cancer

#### 2.4.1 Functional characteristics and clinical features of short-chain L-3-hydroxyacyl-CoA dehydrogenase in colon cancer

Unlike gastric, kidney, and liver cancers, HADH is highly expressed in colon cancer cells (Ren et al., 2020). High HADH levels can promote colon cancer cell proliferation and are significantly associated with poor clinical outcomes (Voloshanenko et al., 2018; Ren et al., 2020). Thus, HADH potentially functions as an oncogene in colon cancer.

#### 2.4.2 Signaling pathways influenced by short-chain L-3-hydroxyacyl-CoA dehydrogenase in colon cancer

In colon cancer cells, the Wnt signaling pathway is required for HADH-mediated regulation of cell proliferation (Voloshanenko et al., 2018). Wnt signaling is involved in a variety of events throughout embryonic development and tissue homeostasis, and has also been linked to cancer (Mao et al., 2014; Kahn, 2014; Morin et al., 1997; Clevers and Nusse, 2012; Roelink et al., 1992; Clements et al., 2002; van’t Veer et al., 1984; Cleary et al., 2014). β-catenin-dependent (canonical) and independent (non-canonical) signaling are two types of Wnt signaling (Zhan et al., 2017). Multiple intracellular signal cascades can be triggered by Wnt ligands, which can orchestrate complicated context-dependent responses. With the aid of Porcupine (Porcn) and Evi/Wls/GRP177, cells can release Wnt ligands in an autocrine or paracrine manner (Kadowaki et al., 1996; Herr and Basler, 2012). Wnt5a/b has been demonstrated to regulate HADH expression, with HADH relying on Evi/Wls secretion to act on the β-catenin-independent Wnt signaling pathway for regulation of colon cancer cell growth and proliferation (Voloshanenko et al., 2018). Dvl2 and RoR2 are also involved in the regulation of HADH, which is consistent with existing knowledge of the participation of RoR2/Dvl2 in β-catenin-independent Wnt signaling (Boutros et al., 1998; Nishita et al., 2010; Ishida-Takagishi et al., 2012) (Figure 3). ATF2 and ATF4 transcription factors are also involved in regulating HADH.

### 2.5 Acute myeloid leukemia

#### 2.5.1 Functional characteristics and clinical features of short-chain L-3-hydroxyacyl-CoA dehydrogenase in acute myeloid leukemia

Similar to colon cancer tissues, HADH expression was markedly upregulated in acute myeloid leukemia patient samples. Wei et al. showed that HADH upregulation was significantly associated with poor OS (Wei et al., 2020).

#### 2.5.2 Signaling pathways influenced by short-chain L-3-hydroxyacyl-CoA dehydrogenase in acute myeloid leukemia

Glutathione peroxidases (GPXs) are peroxidase enzymes that reduce lipid hydroperoxide and free hydrogen peroxide levels to protect organisms from oxidative damage (Takebe et al., 2002). In mammals, eight GPX sub-members have been discovered (Margis et al., 2008), which have been reported to play key roles in repairing reactive oxygen species (ROS)-induced damage, shielding DNA, proteins, and lipids from oxidative damage (Brigelius-Flohé and Maiorino, 2013), and carcinogenesis (Peng et al., 2014; Yang et al., 2014; Nalkiran et al., 2015; Chen Z. et al., 2017; Hangauer et al., 2017; Jiao et al., 2017; Liu et al., 2017; Viswanathan et al., 2017; An et al., 2018; Metere et al., 2018; Naiki et al., 2018; Zhu et al., 2018; Zhou et al., 2019a; Zhou et al., 2019b; Cai et al., 2019; Cheng et al., 2019; Lin et al., 2019; Wang et al., 2019; Yi et al., 2019; Li et al., 2020). Wei et al. discovered that HADH expression was linked to GPX-7 and RPP40 (Figure 3). However, the precise molecular mechanism by which HADH acts in acute myeloid leukemia is unknown, and more research is needed.

## 3 Conclusion and future perspectives

Around the world, cancer incidence and mortality are quickly rising. High-throughput gene expression profiling technologies allow for the simultaneous screening of expression levels of thousands of genes. Identifying variations in gene expression patterns between tumor and control samples is one of the key goals of gene expression profiling in cancer (Feten et al., 2007). Technological advancements and less expensive DNA sequencing procedures have fueled global efforts to identify relevant differentially expressed genes. In various human cancers, including gastric cancer, kidney renal clear cell carcinoma, liver cancer, colon cancer, and acute myeloid leukemia, the recently discovered gene HADH was found to be widely elevated or downregulated depending on the disease. Extensive therapeutics that target HADH have yet to be produced, leading to potential future developments. Multiple clinicopathological characteristics and patient prognoses were significantly associated with HADH expression levels, including clinical stage, histologic grade, immune cell infiltration, OS, DFS, and distant metastases. *In vitro* investigations have demonstrated that HADH can influence tumor cell proliferation, migration, and invasion rates in numerous malignancies, supporting its role in carcinogenesis and tumor progression. Preliminary findings reveal that HADH can impact multiple signaling pathways that promote carcinogenesis and cancer progression, including AKT, Wnt, EMT, TNF-α, IL-6-JAK-STAT3, and interferon signaling pathways.

Although HADH is a potential therapeutic target, several questions still remain to be addressed. Firstly, the molecular mechanisms of HADH in different types of cancers are not completely understood. Previous studies have suggested that HADH serves as a tumor suppressor gene in gastric cancer, kidney renal clear cell carcinoma, and liver cancer by inhibiting cell proliferation, migration, and invasion, as well as being associated with cancer progression and patient survival. However, it can also exist as an oncogene in colon cancer and acute myeloid leukemia, where it promotes cell proliferation and is associated with poor patient outcomes. Pathway analyses of HADH activity have only been conducted in kidney renal clear cell carcinoma, liver cancer, colon cancer, and acute myeloid leukemia, but the specific molecular mechanisms were not explained in detail. Furthermore, while the functions of HADH in gastric cancer, kidney renal clear cell carcinoma, liver cancer, colon cancer, and acute myeloid leukemia have been studied to some extent, its potential role in other cancers, such as cancers associated with the respiratory and reproductive systems, remain unexplored. Secondly, the search for diagnostic biomarkers or therapeutic targets is a promising direction for cancer diagnosis and treatment. HADH can be upregulated or downregulated in certain tumor tissues, but it is currently not known if HADH is also upregulated or downregulated in body fluids such as urine and plasma. Next, we will focus on whether the levels of upstream and downstream factors in the HADH pathway are changed in urine and serum, as well as further analyze whether expression of HADH itself changes in these body fluids. If HADH is detected in urine or plasma, a simple non-invasive test can possibly be performed to use HADH as a cancer-specific molecular biomarker to facilitate early detection and prognostic assessment of specific cancers. Thirdly, whether HADH can play a role in cancer diagnosis as a tumor-associated antigen is still unknown and requires further evaluation. Lastly, HADH is an immune system-associated gene, but whether it can play a role in clinical trials and individualized treatment of various immune modulators remains to be seen. Therefore, more attention should be paid to the clinical value of HADH in cancer diagnosis and treatment.

In summary, various studies have shown that HADH can have oncogenic or tumor suppressive functions in cancer development and progression and may serve as a potential cancer-specific molecular biomarker in the diagnosis, treatment, and prognosis of different types of cancers. Some progress has been made in studying the mechanism of HADH, but this work is currently in early stages. Future investigations should focus on exploring the precise molecular mechanism of how HADH is regulated in carcinogenesis and tumor progression to support its potential clinical application.

## Abbreviations

HADH, short-chain-L-3-hydroxyacyl-CoA dehydrogenase; TME, tumor microenvironment; FAO, fatty acid oxidation; TIICs, tumor-infiltrating immune cells; PTEN, phosphatase and tensin homolog; AKT, serine threonine kinase; EMT, epithelial-mesenchymal transition; TNF- α, tumor necrosis factor-α; IL-6, interleukin 6; JAK, Janus kinase; STAT3, signal transducer and activator of transcription 3; ROR2, receptor tyrosine kinase like orphan receptor 2; Dvl2, disheveled segment polarity protein 2; ATF2, activating transcription factor 2; ATF4, activating transcription factor 4; GPX-7, glutathione peroxidase 7; Rpp40, ribonuclease p protein subunit p40; OS, overall survival; DFS, disease-free survival; HCC, hepatocellular carcinoma
